# Current status, challenges and the way forward for clinical pharmacy service in Ethiopian public hospitals

**DOI:** 10.1186/s12913-017-2305-1

**Published:** 2017-05-19

**Authors:** Arebu Issa Bilal, Zelalem Tilahun, Gebremedhin Beedemariam Gebretekle, Belete Ayalneh, Bisrat Hailemeskel, Ephrem Engidawork

**Affiliations:** 1 Departement of Pharmaceutics and Social Pharmacy, School of Pharmacy, College of Health Sciences, Addis Ababa University, Addis Ababa, Ethiopia; 2 Departement of Pharmacology and Clinical Pharmacy, School of Pharmacy, College of Health Sciences, Addis Ababa University, P.O. Box: 1176, Addis Ababa, Ethiopia; 3 Deprtment of Pharmacy Practice, College of Pharmacy, Howard University, Washington DC, USA

**Keywords:** Clinical pharmacists, Clinical pharmacy service, Job satisfaction, Graduates, Public hospitals, Ethiopia

## Abstract

**Background:**

Clinical pharmacy service has evolved steadily over the past few decades and is now contributing to the ‘patient care journey’ at all stages. It is improving the safety and effectiveness of medicines and has made a significant contribution to the avoidance of medication errors. In Ethiopia, clinical pharmacy service is in its initial phase, being started in July 2013. This study therefore aimed at assessing the status, challenges and way forward of clinical pharmacy service in the country.

**Methods:**

A cross-sectional survey was conducted in six regional states and one city- administration in September 2014. A total of 51 hospitals were included in the study. Both qualitative and quantitative methods were employed for data collection.

**Results:**

A total of 160 pharmacy graduates, and 51 pharmacy heads participated in the study. Internal Medicine and Pediatric wards were the major wards where the graduates provide clinical pharmacy service. Almost 94% of the new graduates were found to be involved in clinical pharmacy service, but 47% of them rated their service as poor. The overall satisfaction of the graduates was close to 36%. Thirteen hospitals discontinued and two hospitals not even initiated the service largely due to shortage of pharmacists and lack of management support. About 44% of the surveyed hospitals documented the clinical pharmacy service provided using either developed or adopted formats. Lack of awareness by the medical fraternity, high attrition rate, lack of support from the management as well as from the health care team, readiness of the graduates to deliver the service, and shortage of pharmacists were identified by the key informants as the major stumbling block to deliver clinical pharmacy service.

**Conclusion:**

Clinical pharmacy service is initiated in most of the surveyed hospitals and a large proportion of the graduates were involved in the service. Although there is a great enthusiasm to promote clinical pharmacy service in the surveyed hospitals, efforts made to institutionalize the service is minimal. Thus, concerted efforts need to be exerted to promote the service through organizing awareness forums as well as revisiting the curriculum.

## Background

The mission of the pharmacy profession is to improve public health through ensuring safe, effective, and appropriate use of medications [1]. Pharmaceutical service is considered as the backbone of the entire health care system as patients do not usually visit health facilities where there are no medicines. Thus, availability of medicines and a competent pharmacy workforce is therefore crucial for the well-functioning of the pharmaceutical service [2].

According to the Global Pharmacy Workforce Survey conducted between September 2011 and June 2012 [3], African countries were demonstrated to have a low density of pharmacists as well as pharmacies, indicating limited access points for medicines provision and skilled human resources for their management. The survey ranked Ethiopia as the 9^th^ country among countries with the least number of pharmacists. Despite this shortage, there is a global trend of moving towards pharmaceutical care over the past four decades, which enables pharmacists to assume an expanded role and necessitates reforms in pharmacy education and practice [4]. This global shift requires an integrated academia for training and a competent and sufficient workforce that would assume the new roles [5, 6].

Currently, the clinical pharmacy service is initiated in various countries. This area of practice is, however, at its infancy in Ethiopia. Ethiopia has been known for its long track record of product-oriented pharmacy practice. Recently, there is a shift towards a patient-focused practice following development and implementation of a 5-year Bachelor of Pharmacy (B.Pharm) curriculum (which includes a one-year clerkship program) [7, 8]. Even though few masters’ graduates in clinical pharmacy and in-service trained pharmacists started clinical pharmacy service in few public hospitals in the country, the service was officially launched in most of the public hospitals in September 2013, when the first 426 graduates of the new curriculum came out in July 2013 [8, 9]. These graduates were immediately deployed in public hospital settings found in different regions and city administration of the country by the Federal Ministry of Health (FMoH), with the ultimate aim of initiating or expanding clinical pharmacy service. Higher education training in Ethiopia involves cost-sharing scheme, where 15% of the cost is borne by students. This cost is, however, covered by the government, with a condition that students serve the public sector after graduation for five years. Otherwise, they should pay the 15% cost and will be free to serve in any sector.

The Ethiopian pharmacy sector is experiencing a new initiative. Thus, there is a need to carry out an assessment on successes and challenges of the service provided by the new graduates so that both the curriculum and the practice could be informed. The main aim of this study was therefore to assess the status of clinical pharmacy service in selected Ethiopian public hospitals following deployment of the new pharmacy graduates.

## Methods

### Description of the study area

Ethiopia is located in the Eastern part of Africa and has a total surface area of 1.1 million square kilometer. Administratively, the country is divided into nine National Regional States (Afar, Amhara, Benishangul Gumuz, Gambella, Harrari, Oromia, Somali, Southern Nations Nationalities and Peoples (SNNP) and Tigray) and two City Administrations (Addis Ababa and Dire Dawa). According to Central Statistical Agency’s population projection, the population of Ethiopia was expected to reach 90.1 million in 2015; of which 45.3 million were males and 44.8 million were females [10]. The country has made several health reforms and initiation of clinical pharmacy service is one of the activities that has received attention by the policy makers. Ethiopia has functional 16,447 Health Posts, 3547 Health Centers, and 189 Hospitals [11]. Although recent data is not available, previous studies indicated that the country had had 1989 pharmacists and the distribution across regions was very much skewed [12].

### Study design

A cross sectional survey was conducted in six regional states and one City administration in September 2014. Concurrently, a phenomenological study employing an in-depth interview was done with pharmacy heads of the selected hospitals.

### Study population

New graduates from the public schools of pharmacy deployed by the FMoH in 2013 and working in public hospitals, and Heads of Pharmacy service of the selected health institutions formed the study population.

### Inclusion and exclusion criteria

Of the 9 regions and 2 city administrations, six regions (Amhara, Harari, Oromia, SNNP, Somali, and Tigray) and one city administration (Addis Ababa) were selected purposively, taking into account the number of new graduates deployed. The other regions (Afar, Gambella, Benshangul-Gumuz and Dire Dawa) were excluded from the study as they had only one or two new graduates per region. From the selected regions and city administration, all hospitals that had employed the new pharmacy graduates were included in the survey. Accordingly, those new pharmacy graduates and Heads of the Pharmacy service working in the 32 General hospitals, 14 Referral hospitals and 5 specialized hospitals were included in the study. All new graduate pharmacists available at the time of data collection were included in the study. In addition, an in-depth interview was made with 51 Heads of the Pharmacy service of the selected hospitals.

### Data collection procedure

Quantitative data was collected using a pre-tested and self-administered questionnaire customized based on previous studies. Qualitative data was obtained using semi-structured, open topic guides with flexible probing technique. Different variables of interest were used to assess the status and challenges, how to overcome the challenges, appropriateness of the setup, and the type of service provided in the hospitals. A total of 12 pharmacy postgraduate students were recruited as data collectors. They attended a one day training to familiarize them with the survey questionnaire and guiding questions for the key informant interview. Besides the practical training, adequate supervision and follow up was done by the supervisors to maximize quality of the data collected. All interviews were audio recorded and this was supplemented by note taking during the interview. The local language (Amharic) was used during the interview and duration of the in depth interview was approximately thirty minutes.

### Data analysis

The quantitative data was coded, entered, cleaned and analyzed using SPSS version 20. Descriptive statistics was used for summarizing results in tables and graphs. Analysis of the qualitative data involved an intensive reading through the data to identify key themes. Satisfaction rate was rated using a Likert scale ranging from strongly agree (5 pts) to strongly disagree (1 pt.). For purpose of analysis, strongly agree and agree were categorized as “Agree”, while strongly disagree and disagree as “Disagree”. All notes and the transcribed audio records were translated into English. The transcripts were coded and thematic analysis was used to analyze the data. To check accuracy of the translation, one of the recordings was translated and transcribed by a bi-lingual pharmacist and compared with the primary work. Furthermore, findings of the study were communicated to five of the participants who were randomly selected for approval of the transcripts and interpretations.

### Ethical consideration

Ethical approval was sought from the Ethics Review Committee of the School of Pharmacy, Addis Ababa University (Ethical approval letter no ERB/SOP/01/07/2014). Permission was also granted from the selected health facilities and verbal informed consent was obtained from each participant. Participants were informed that they could withdraw from the study at any time. The privacy of participants was fully respected during data collection and sessions were arranged in a private and quiet place. Identifiers were not used to ensure anonymity and the audio records as well as transcripts were kept in a safe place, and were accessible to the research team.

## Results

A total of 160 graduates, which accounted for 37.5% of the total graduates, of the new curriculum working in the 51 hospitals were included in the survey. Relevant information on socio-demography and practice sites of the survey participants is summarized in Table 1. Majority of the graduates (77.5%) were males and almost all (99.99%) were single. Mean age of the participants was 24.78 years with a standard deviation of (SD ± 1.26). The most common practice sites were internal medicine ward (46%) and outpatient dispensary (46%) followed by pediatrics ward (24%) and Drug Information Center (DIC) (17.0%). On average, each graduate had 12.21 ± 1.85 months of experience and more than 91% of them had ≥ 12 months experience in a hospital setting.

**Table 1:** Socio-demographic characteristics of pharmacy graduates in Ethiopian Public Hospitals, September 2014

Variables		*N* (%)
Gender	Male	124(77.5)
Marital status	Single	159(99.0)
Practice sites	Internal medicine ward	73(46.0)
	Dispensary	73(46.0)
	Pediatrics ward	38(24.0)
	DIC	27(17.0)
	Surgery ward	9(6.0)
	Emergency ward	3(6.0)
	Others^a^	10(6.0)
Years of work experience	≥ one year	146(91.0)
	< one year	14(9.0)

*n* = 160; Others^a^: Psychiatry, Oncology/Hematology, Gynecology/Obstetrics wards

### Implementation status of clinical pharmacy service

Although over 90% of the participants reported to have been involved in the provision of clinical pharmacy service to patients, a considerable number of them (47%) rated their service as poor. The two top reasons were lack of support from hospital management and lack of incentives or recognition, each accounting for over 80%. Other most common reasons were lack of support from the health care professionals, lack of support from the pharmacy case team, and high work load (Table 2).

**Table 2:** New Pharmacy graduates’ possible reasons for rating their service as poor, September 2014

Possible reasons	*N*(%)
Lack of support from Hospital management	62(88.57)
Lack of incentives and/or recognition	57(81.43)
Lack of support from other health care professionals	38(54.29)
Lack of support from pharmacy case team	37(52.86)
High work load	32(45.71)
Lack of interest to provide clinical pharmacy services	7(10)
Lack of skills (communication, counseling and technical)	6(8.57)
Lack of knowledge	4(5.71)
Lack of confidence	2(2.86)
Others^a^	2(2.86)

*n* = 70; Respondents gave multiple responses and percentages will not add up to 100%

Others^a^: Lack of in service training, poor government commitment

### Level of job satisfaction

Graduates alluded to the fact that inter-professional collaboration with physicians, nurses and health officers (Health care professional with a Bachelor degree in Public Health) would have a paramount importance for better service delivery. From the total of seven items used to assess inter-professional collaboration, greater proportion of the respondents said that they were satisfied with such collaboration (Table 3).

**Table 3:** Clinical pharmacists level of job satisfaction in clinical pharmacy service, September 2014

Items	*SA=strongly agree, A=agree, N=Neutral, DA=Disagree, SDA=Strongly Disagree, SD=Standard Deviation; Responses ranged from strongly agree (5) to strongly disagree (1)*			
	Agree *N* (%)	Neutral *N* (%)	Disagree *N* (%)	Mean ±SD
Items related with inter professional collaboration				
Physicians consult me on professional matters	83 (51.9)	40 (25)	37(23.2)	3.28±1.1
Physicians cooperate when I communicate “job-related” matters with them	116 (72.5)	23 (14.4)	21 (13.2)	3.67±0.95
Health officers consult me on professional matters	67 (41.9)	59 (36.9)	33 (20.6)	3.21±1.01
Health officers cooperate when I communicate “jobrelated” matters with them	71 (44.4)	70 (43.8)	19 (11.9)	3.34±0.91
Nurses cooperate when I communicate “job-related” matters with them	122 (76.2)	24 (15)	14 (8.8)	3.86±0.9
Nurses often initiate consultations with me on professional matters	106 (66.2)	30 (18.8)	24(15.0)	3.67±1.0
I am satisfied with the “on-the-job” relationships I have with others	80(50.0)	44 (27.5)	36 (22.5)	3.34±1.03
Items related with service recognition				
The working environment is conducive	61(38.2)	31 (19.4)	68(42.5)	2.86±1.17
The hospital management respects and treats pharmacy professionals similar to other health professionals in the hospital	52 (32.5)	32 (20.0)	76 (47.5)	2.71±1.25
The acceptance of clinical pharmacy services in the hospital is good	72 (45.0)	44 (27.5)	44 (27.6)	3.21±1.15
Staff working with me treat me with professional respect	115 (71.9)	36 (22.5)	9(4.7)	3.81±0.83
The services that I provided is recognized by the community	42 (26.3)	48 (30.0)	70 (43.7)	2.61±1.2
My salary is appropriate	12(7.6)	24 (15.0)	124 (77.5)	1.81±0.99
Items related with commitment and professional satisfaction				
My talents are fully utilized on my job	55(34.1)	33 (20.6)	72 (45.1)	2.82±1.17
I like spending the rest of work life in my current job	55 (34.4)	33 (20.6)	72 (45.0)	2.76±1.37
Time goes quickly while I am at work	89(55.7)	36 (22.5)	35 (21.9)	3.41±1.19
I often leave work with a feeling that I’m doing something which I enjoy	66 (41.3)	36 (22.5)	58 (36.3)	2.98±1.25
If I had to decide all over again, I would still choose pharmacy again	71 (44.4)	29 (18.1)	60 (37.5)	2.99±1.5
If my children are interested in pharmacy, I will encourage them to pursue it as a career	72(45.1)	31 (19.4)	57 (35.7)	3.01±1.43
All things considered, I am satisfied with my job	57 (35.7)	29 (18.1)	74 (46.2)	2.78±1.29

*n* = 160

Among the six items used to assess satisfaction in service recognition, gaining professional respect by other professionals was considered as the major item for satisfaction by about 72% of the respondents. On the other hand, salary scale was identified to be the main cause of dissatisfaction by over three-quarter (77.5%) of the respondents. Satisfaction in terms of commitment and interest was also evaluated using seven items for rating. There seemed to be no major variation in the proportion of respondents who agree and disagree in majority of the items. However, a sizable percentage of the respondents said that time went by quickly while they were at work.

### Suggestions related to the undergraduate curriculum

**Fig. 1:**
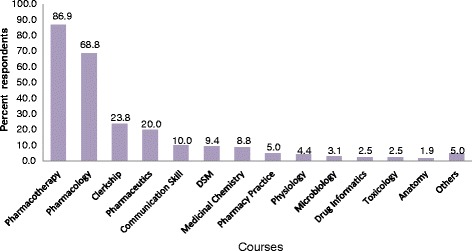
Undergraduate courses identified by respondents based on their contribution to the practice, September 2014, Ethiopia: Respondents gave multiple responses; Others: pharmaceutical calculation, pharmacogenetics, industrial pharmacy, biochemistry, medical supplies, Pharmacy law & ethics

**Fig. 2:**
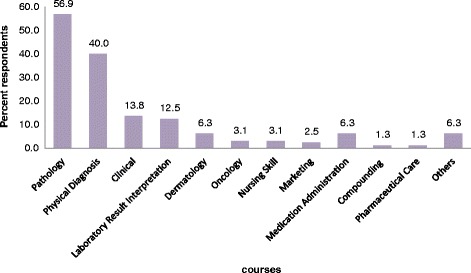
Clinical pharmacists’ suggestion for courses to be included in the undergraduate curriculum, September 2014, Ethiopia: Respondents gave multiple responses; Clinical courses (internal medicine, surgery, pediatrics, and gynecology/Obstetrics); others: computer, emergency medicine, radiology, psychiatry, hematology and parasitology

The respondents identified courses that would help them for practice. Among these, pharmacotherapy (87%), pharmacology (68.8%), and clerkship (23.8%) were the most commonly cited ones (Fig. 1). They also suggested courses that should be included in the current curriculum (Fig. 2) to further improve their clinical knowledge. The courses are pathology (56.9%), physical diagnosis (40%) and clinical courses (13.8%). The clinical courses included internal medicine, pediatrics, surgery, and Gynecology/Obstetrics. Revisiting credit hour allotted to the different courses was one issue the participants raised during the survey. Credit hour increase was proposed for courses including, anatomy, physiology, clerkship, pharmacology, pharmacotherapy, and microbiology.

The graduates also forwarded several recommendations to improve the pharmacy education and service. Promotion and recognition of the service was the major recommendation made by about 40% of the respondents (Table 4). Other useful recommendations related to the curriculum, career path, and benefits had also been forwarded.

**Table 4:** Suggestion forwarded to improve clinical pharmacy education and service in the future, September 2014

Suggestions	Frequency^a^	Percentage (%)
Promotion and recognition of clinical pharmacy service	66	41.0
Increase salary and other benefits	47	29.0
Starting the clerkship early	35	22.0
Providing in-service training for clinical pharmacists	32	20.0
Revisiting the MSc and/or launching PharmD program	23	14.4
Providing appropriate facilities	22	14.0
Preparing clear Job description	20	12.5
Revising the curriculum	15	9.4
Using annual based course allocation	7	4.4
Building instructors capacity	7	4.4
Government support and commitment	7	4.4
Preparing standard documentation forms	5	3.0
^b^Others	12	7.5

^a^Respondents gave multiple responses

^b^formulation of medico-legal sanctions and separating clinical and industrial pharmacy

### Qualitative findings

Out of the 51 hospitals surveyed, 13 hospitals discontinued the clinical pharmacy service and 2 hospitals did not start the service at all. The main reasons for cessation of the clinical pharmacy service were shortage of pharmacists in the dispensary, disagreement with physicians, inappropriate set up, lack of hospital management support, lack of clear job description and high attrition rate. The clinical pharmacy service was found to be better in those hospitals where the service was previously initiated by the MSc graduates or in-service trained pharmacists.

In hospitals where clinical pharmacy service was provided, graduates were actively involved in the following activities: attending morning sessions and rounds, patient follow up, providing current drug information, assisting in drug treatment selection, medication errors containment, drug reconstitution, and documentation and preparing patient leaflets. However, the survey revealed that only 22 of the surveyed hospitals were found to document the service being provided. Some of them used formats prepared by Management Sciences for Health/MSH/SIAPS for service documentation, while others used in-house developed documentation formats. As the respondents explained, in few hospitals (*n* = 5), the rendered clinical pharmacy service would be periodically reported to the Regional Health Bureau and Pharmaceutical Fund and Supply Agency (PFSA).

### Challenges of implementation of Clinical Pharmacy Services

A multitude of factors were found to affect the implementation of clinical pharmacy service. These factors were: (i) Pharmacist related factors, (ii) Other health care professionals’ related factors, and (iii) Hospital management related factors. Each sub-theme is reviewed in detail as follows.

i) Pharmacists related factors

Clinical knowledge gap, high attrition rate, negligence (no self-updating), lack of interest and confidence to discuss with physicians, poor commitment and motivation, and poor communication skills were the most common deterring factors reported by the pharmacists. The problem might even be worse, since there was no special retention mechanism put in place in majority of the hospitals. Most of the graduates appeared to stay in the hospitals because they could not get a release paper so as to work in other sector, such as the private sector. Nevertheless, there were few hospitals that motivated the graduates by providing personal computers and by allowing duty payments.

#### High attrition rate

Findings from this study revealed that at least one graduate had left from 12 of the surveyed hospitals. Attrition was invariably identified by the respondents as the major challenge for the sustainable provision of clinical pharmacy service in the hospitals. One pharmacy service head described the problem as follows:

*“High attrition rate of the graduates is the main reason why clinical pharmacy service in our hospital is compromised” (Specialized hospital, Participant # 35).*

Another *pharmacy head* said that:

*“The clinical pharmacy service was blossoming in our hospital with deployment of the graduates. Everybody, specially, physicians were acknowledging contributions of the graduates. However, the service started to dwindle and then discontinued after they had left” (General hospital, Participant # 38).*

The main reasons for attrition as mentioned by the respondents were: lack of interest to work in the dispensary, little acceptance by physicians and seeking better pay.

One of the pharmacy head narrated the case as follows:

*“The low level of acceptance and the desire for better payment made the graduates leave their job” (General hospital, Participant # 38).*

Another pharmacy head also expressed the condition as follows:

*“The lack of interest to work in the dispensary forced the graduates leave their job” (General hospital, Participant #33).*

#### Lack of interest, confidence and poor communication skill

Majority (*n* = 35) of the key informants indicated that there is lack of interest and commitment as well as lack of confidence and poor communication skill to provide clinical pharmacy service.

One of the pharmacy head narrated the condition as follows:

*“It’s been very difficult to sustain the service. Lack of motivation, commitment and energy to face existing problems by fellow graduates is the major setback in our hospital* (*Referral hospital, Participant #10)”*).

Another *pharmacy head* also said that:

*“There is poor communication between the graduates and other health care professionals. I think the graduates are not confident enough and I believe they do not have adequate clinical knowledge to communicate with other health care professionals in the ward”(Referral hospital, pharmacy head # 19)”).*

This has been strengthened by another *pharmacy head*:

*“Most of the graduates are not confident enough to initiate discussion with physicians. They don’t use a systematic approach to communicate with physicians”. (Specialized hospital, Participant #26).*

ii) Other health care professionals related factors

According to the key informants (*n* = 23), lack of awareness and poor attitude towards the clinical pharmacy service, reluctance to accept the pharmacists in the health care team, and lack of proper communication were among the factors that negatively contributed to the growth of the service. One pharmacy head expressed the condition as follows:

*“……Majority of the health care professionals do not have awareness to the clinical pharmacy service, as it is new not only to our hospital but also to the nation. Besides, during my informal communication with some prescribers, they expressed their fear that their wrong-doing in prescribing will be disclosed, since they perceive the pharmacists as fault finders (Referral hospital, Participant #27).*

A similar concern was also raised by another Pharmacy head;

*“Pharmacists believe that the physicians’ perceive them as “fault finders” and are hesitant to accept the pharmacists in the clinical practice (Specialized hospital, Participant #26).*

iii) Hospital management related factors

The study participants (*n* = 40) identified absence of clear job description/scope of practice, lack of additional benefits and incentives, lack of proper monitoring and supervision, lack of standard documentation formats, poor hospital management support, lack of appropriate setup for the service, and absence of pre-established system in the hospital as a hospital management related challenges for implementing clinical pharmacy service.

One of the pharmacy heads described the circumstance like this;

*“The graduates have been doing a great job, but their service was not recognized and appreciated by the hospital management. Although the service is a continuous care to be provided at all times like the medical service, the hospital management refused to pay duty payment for those assigned in the wards” (General hospital, Participant #23).*

Another participant also mentioned that:

*“Lack of pre-established system in the hospital made the service provision difficult as per the standard. Moreover, the management does not have a clear view about the service. The graduates do not have a room to read and even to change their clothes in the wards. In general, the setup of the hospital is not conducive for the professionals” (Specialized hospital, Participant #35).*

It was also learnt that the implementation of Auditable Pharmacy Transaction Service (APTS) in few hospitals somehow affected the service. Although APTS is a tool that improves supply management in a hospital, it is known to be labor intensive. The launch of this system led to channeling of the workforce from the ward to the dispensary. As regards to the curriculum, around a third of the participants (*n* = 15) said that the curriculum should be amended so that it could be fit for practice. Indeed, one of the participants expressed his view like this:

“*The new curriculum is not producing confident pharmacists to provide clinical pharmacy service and hence it needs amendment”…referral hospital, pharmacy head…05*.

### The way forward to improve clinical pharmacy service

The following gaps were identified that should be addressed in order to improve the contribution of clinical pharmacy service in the Ethiopian health care system; (i) Promotion and awareness creation of clinical pharmacy service, (ii) Curriculum related, (iii) Pharmacy work force related, (iv) Guidelines and formats related, (v) Salary and incentives related, and (vi) Appropriateness of the setup for service.

i) ***Promotion and awareness creation of clinical pharmacy service***

All of the key informant interview respondents (*n* = 51) agreed that adequate promotion and awareness creation about the service had not been done. This was evidenced from the script of one of the respondents:

*“The health care professionals, hospital administration staff members and other stakeholders were not well aware about the new clinical oriented pharmacy curriculum and the clinical pharmacy service. It is therefore imperative to use all available means to promote their role and maximize their contribution. (Referral hospital, Participant #28).*

ii) ***Curriculum related issues***

Majority of the pharmacy heads (*n* = 40) stated that the curriculum should be revised so that it could improve practical skills of graduates. They alluded to the fact that non-clinical courses should be minimized so as more room would be available for clinical courses in the curriculum. In addition, they also echoed that early exposure of students to the experiential training in the clinical setup is mandatory to improve their skill. The other issue raised by the respondents was about the need for national standardized curriculum on the theoretical and practical training. This idea was raised because their observation on the knowledge and skill differences of graduates. One of the participants articulated the difference as follows:

“*The graduates who came from the older Universities have better performance as compared to the new ones” (Referral hospital, Participant #03)*.

In addition, staff development plan, postgraduate curriculum assessment, continuing education program designing, and in service training were other recommendations forwarded in the area of education. This was highlighted as shown below by one of the respondents:

*“Continuous follow up and in service training are important for the betterment of the service in the country”(Referral hospital, Participant #18).*

iii) **Pharmacy workforce issues**

The respondents invariably agreed on the shortage of clinical pharmacists in particular and pharmacy professionals in general in their respective hospitals. One of the pharmacy head expressed his view in the following way:

“*The regional health Bureau is willing to recruit additional pharmacists when we ask them. However, the limit posed on the number of pharmacists assigned in each level of the health institution by the Ministry of Civil Service is a bottleneck. There is obviously a huge demand of pharmacists in our hospital as a result of initiation of APTS and clinical pharmacy service”. (Referral hospital, Participant #20)*.

Moreover, respondents reminded fellow clinical pharmacists to work with motivation and dedication in the wards so as to improve patient outcome.

iv) **Guidelines and formats**

Majority of the respondents (*n* = 47) also stated that the concerned authorities should prepare clear job description, formulate evaluation guidelines, design monitoring and evaluation system, and prepare standard documentation formats.

One pharmacy head mentioned the scenario like this:

*“There is no clear job description. This is one of the major problems that we are facing in the implementation process. Hence, concerned authorities should prepare an appropriate job description for the graduates”. (Referral hospital, Participant #11).*

Another respondent expressed the documentation problem as follows:

*“Patient charts used in the hospital do not have a place where pharmacists write their recommendations/interventions. This has created problem in terms of accountability”. (General hospital, Participant #8).*

v) **Salary and incentives**

The participants stated that the new graduates are de-motivated by their salary and absence of additional benefits (*n* = 45). They recommended that the government should look into this aspect of the problem.

This was expressed by respondents’ as follows:

*“Even if the graduates are working hard in the ward, they don’t have any additional benefits. The management should explore ways to incentivize the graduates”. (Specialized hospital, Participant #29).*

*“The graduates are disappointed by the low salary as well as lack of incentive packages” (General hospital, Participant #33).*

vi) **Appropriateness of the setup for service**

Participants from Addis Ababa stated that the hospital setup is appropriate to implement clinical pharmacy service, since most of the hospitals are referral and specialized hospitals having different inpatient departments. However, inaccessibility of office, computers, internet, reference materials and other facilities were cited as challenges to implement the service. On the other hand, most of the respondents (*n* = 30) out of Addis Ababa said that the setup is not appropriate for implementation of the service. They went on saying that this is due to small number of specialties in district hospitals and poor support of hospital management for the service. Besides, they shared the problems mentioned above by participants from Addis Ababa and indicated that the problem is much more grave in their settings..

This was clearly narrated by one of the respondent as described below;

*“Overall, the setup is not appropriate for the service. Clinically oriented Pharmacists have no room and furniture as well as references they consult for rendering a better service”. (Specialized hospital, Participant #21).*

## Discussion

This study attempted to give an insight in the implementation of clinical pharmacy service in the public hospitals of Ethiopia. Apart from assessing strength and limitations of the service, it also sheds light on the level of job satisfaction of newly graduated pharmacy professionals practicing in the surveyed hospitals.

Most of the new graduates were working in internal medicine and pediatrics wards. This may be due to the high prevalence of drug related problems in the internal medicine ward and vulnerability of the patient population in the pediatric ward. The graduates were also assigned in the dispensary due to shortage of pharmacists, which was compounded by implementation of workforce intensive initiatives. This in turn affected the clinical pharmacy service expected to be provided in the wards. Assigning more pharmacists in the different wards could have a significant impact on the provisions of clinical pharmacy service. Assignment of little or no pharmacists in wards, such as Surgery, Gynecology and Obstetrics, and Psychiatry strongly indicates the need for deployment of more graduates. As mentioned by the key informants and other studies [5, 13], increasing presence of clinical pharmacists in such wards is crucial to increase patient outcomes. The workload that was borne by the graduates would obviously undermine their performance in the ward and thus Ministry of Health and hospital management should look into possibilities of recruiting more graduates.

Even if our previous study done in similar settings reported that large proportion of the health care providers had positive attitude towards clinical pharmacy service [8], the graduates said that they did not have adequate support from health care professionals as well as from the management. It has been shown that collaboration among the various healthcare professionals could lead to significant improvement in patient care [14]. Thus, hospitals in Ethiopia should create a platform, where inter-professional collaboration could be fostered. It is also evident that without sufficient support from the management side, the program will not move forward to meet its intended goals. Hence, there is a need for continuous discussion and engagement of other professionals and the management during the inception and implementation phases of the initiative so that they could provide the necessary assistance.

Documentation of clinical pharmacy activities and the ability to identify pharmacists’ interventions are essential for assessing their impact on patient outcomes [15]. However, only 44% of the surveyed hospitals documented the clinical pharmacy service rendered to patients. This might be due to the fact that the service is new and there is no well-prepared chart for documenting the service as unveiled by the key informants. This strongly indicates that a system needs to be designed where service rendered could be documented and evaluated as needed. The fact that majority of the respondents were from the male gender is in line with a previous study conducted in Ethiopia, where more than three-fourth of pharmacists in Ethiopia were reported to be males and this variation in gender composition was also evident within the regions and city administrations [12]. Contrary to the present finding, the global workforce has become feminized, with significant countries showing female representation of more than 65% with an increasing trend trajectory [16].

As regards to pharmacists’ level of job satisfaction, the mean value of the response for most of the items was close to 3.0. This is comparable with the study conducted in India that had a mean score of 3.0 [17]. The mean value of “agreement” for the items ranged from 1.81 ± 0.99 for the item “*My salary is appropriate*” to 3.86 ± 0.9 for the item “*Nurses cooperate when I communicate job related matters with them*”. The issue of incentive was again raised by the key informants. This collectively indicates that incentive is an issue that should be addressed and it could probably enhance performance of the graduates. A sizable proportion of the respondents seemed to be satisfied with items related to inter-professional collaboration. One should therefore nurture and cultivate this interaction, as this is the backbone for achieving better patient outcome [18].

Shortage of pharmacy workforce stood out as the major challenge in the present study. Similar findings were also reported in previous studies conducted in Ethiopia [8, 12] as well as elsewhere, such as the UAE [19, 20]. In fact, many countries worldwide have experienced shortage of pharmacists and it is driven not only by the limited resource, but also by the changing role of pharmacists towards pharmaceutical care [6, 21]. In this respect, maintenance and expansion of the pharmacy workforce through implementation of recruitment and retention mechanisms is crucial [18]. Ministry of Health and Ministry of Civil Service should also revise the limit posed in the number of pharmacists assigned in each level of the health institutions*.* Moreover, the need for training more qualified pharmacy professionals that would be able to provide better pharmaceutical care should be given due attention by the academic institutions.

Both from the quantitative as well as the qualitative studies, it was noted that the curriculum should be revised so that it could be fit for practice. Options like minimizing non-clinical courses so as to give more room for clinical courses in the curriculum need to be considered. In addition, early exposure of students to the experiential training in the clinical setup is mandatory to improve their skills.

The study was done shortly after beginning of the implementation of clinical pharmacy service. Thus, contact time could be short to evaluate the clinical pharmacists’ performance. Furthermore, the study involved pharmacy graduates from public universities assigned in public hospitals, and did not cover those from the private sector, so the findings could not be generalized to all pharmacy graduates of the country.

## Conclusion

Clinical pharmacy service is initiated in most of the surveyed hospitals and more than 90% of the newly graduated pharmacists participated in provision of the service. The graduates themselves appeared to be content with the service despite challenges that hindered its expansion. In addition, putting new graduates into challenging situations without adequate support from the management and other health care professionals would jeopardize viability of the service. Shortage of staff, lack of awareness, lack of support from management, hospital setup, incentives, and gaps in the curriculum were some of the challenges identified by all the respondents during the survey. Mitigating these challenges will pave the way for blossoming of clinical pharmacy service in the country.

## Abbreviations

APTS: Auditable pharmacy transaction service
B.Pharm: Bachelor of pharmacy
DIC: Drug information center
MSH: Management sciences for health
PFSA: Pharmaceutical fund and supply agency
SNNP: Southern nations nationalities and peoples
SPSS: Statistical package for the social sciences
